# Chitosan oligosaccharide as a plant immune inducer on the *Passiflora* spp. (passion fruit) CMV disease

**DOI:** 10.3389/fpls.2023.1131766

**Published:** 2023-02-06

**Authors:** Liqun Zhang, Lu Yu, Zhi Zhao, Pei Li, Shuming Tan

**Affiliations:** ^1^ School of Liquor and Food Engineering, Guizhou University, Guiyang, Guizhou, China; ^2^ Qiandongnan Engineering and Technology Research Center for Comprehensive Utilization of National Medicine, Kaili University, Kaili, China

**Keywords:** chitosan oligosaccharide, anti-CMV activity, Passiflora spp., plant immune inducer, brassinosteroids cell signaling pathway

## Abstract

Cucumber mosaic virus (CMV), one of the main viruses, is responsible for *Passiflora* spp. (passion fruit) virus diseases, which negatively affect its planting, cultivation, and commercial quality. In this study, a laboratory anti-CMV activity screening model for *Passiflora* spp. CMV disease was first established. Then, the effects of different antiviral agents of chitosan oligosaccharide (COS), dufulin (DFL), and ningnanmycin (Ning) on CMV virulence rate in *Passiflora* spp. were determined. The virulence rate and anti-CMV activity in *Passiflora* spp. treated with COS were 50% and 45.48%, respectively, which were even better than those of DFL (66.67% and 27.30%, respectively) and Ning (83.30% and 9.17%, respectively). Field trials test results showed COS revealed better average control efficiency (47.35%) against *Passiflora* spp. CMV disease than those of DFL (40.93%) and Ning (33.82%), indicating that COS is effective in the control of the *Passiflora* spp. CMV disease. Meanwhile, the nutritional quality test results showed that COS could increase the contents of soluble solids, titratable acids, vitamin C, and soluble proteins in *Passiflora* spp. fruits as well as enhance the polyphenol oxidase (PPO), superoxide dismutase (SOD), and peroxidase (POD) activity in the leaves of *Passiflora* spp. seedlings. In addition, the combined transcriptome and proteome analysis results showed that COS mainly acted on the Brassinosteroids (BRs) cell signaling pathway, one of plant hormone signal transduction pathway, in *Passiflora* spp., thus activating the up-regulated expression of *TCH4* and *CYCD3* genes to improve the resistance to CMV disease. Therefore, our study results demonstrated that COS could be used as a potential plant immune inducer to control the *Passiflora* spp. CMV disease in the future.

## 1 Introduction

Virus disease, the most common disease in *Passiflora* spp. (passion fruit), could affect the yield and quality of *Passiflora* spp. fruits, thus creating a great threat to the development of *Passiflora* spp. industry (Balendres and Bengoa, 2019; Chen et al., 2021). More than 20 kinds of viruses have been currently identified that could cause *Passiflora* spp. virus disease (Ye et al., 2019). Of which, CMV infection can adversely affect or regulate the phytochemical content of *Passiflora* spp., which will further damage the growth of *Passiflora* spp. (Morales et al., 2002; Yeturu et al., 2017; Mesa et al., 2018; da Cruz et al., 2019; Munguti et al., 2019; Chen et al., 2021; Fu et al., 2021; Huang et al., 2022). However, until now, there are a wide variety of antiviral commercial agents such as chitosan oligosaccharide (COS), dufulin (DFL), and ningnanmycin (Ning) on the market, but the agents mainly targeting virus disease of *Passiflora* spp. caused by CMV have hardly been reported. On another side, the interaction relationship between antiviral commercial agents and *Passiflora* spp. is still vague, resulting in the lack of high-efficiency prevention and control methods.

COS, a chitosan derivative, has been widely applied in diverse fields due to the advantages of low molecular weight, harmless, rapid absorption, and non-toxic stability (Guan and Feng, 2022). Previous studies have shown that COS could promote plant growth, increase the antioxidant enzyme activity, and activate plant resistance (Yuan et al., 2019; Ahmed et al., 2020; Liu et al., 2021; Li et al., 2022a; Yu, 2015). In recent years, COS has been widely applied to control a variety of plant diseases and insect pests, such as *Peronospora parasitica* on Chinese cabbage (Luo and Yan, 2022), wheat powdery mildew (Wu et al., 2022), potato virus Y disease (Yang et al., 2022), kiwifruit soft rot (He et al., 2021), and so on. However, there have been no report on COS used to control the *Passiflora* spp. virus disease caused by CMV.

Proteomics and transcriptome sequencing technologies play a significant role in understanding the protein and gene expression in organism and are one of the best ways to characterize candidate action targets for biological functions (Morozova et al., 2009; Kumar et al., 2016). The difference in protein and gene expression related to biological control process could be identified by the proteomics and transcriptome methods, which are conducive to understanding the mechanism of disease resistance and laying the theoretical foundation for the further prevention and control of viral diseases (Wang et al., 2017; Ji et al., 2019; Wei et al., 2022). Furthermore, a combination of proteomics and transcriptome analysis is greatly helpful to clarify the pathogenicity information and understand the mechanism of action of differentially expressed genes (DEGs) and differentially expressed proteins (DEPs) in related pathways (Kirsch et al., 2012).

In order to find an effective antiviral commercial agent against *Passiflora* spp. CMV disease, in this study, the in laboratory anti-CMV activity screening test and field trials test of COS, DFL, and Ning against *Passiflora* spp. CMV disease were determined. Our results showed that COS is effective in the control of the *Passiflora* spp. CMV disease. Meanwhile, in order to understand the mechanism of action of COS against *Passiflora* spp. CMV disease, the combined transcriptome and proteome analysis was also performed.

## 2 Materials and methods

### 2.1 Model construction of anti-CMV activity test

In June 2021, the branches of *Passiflora* spp., treated with root powder beforehand, were placed in flowerpots with nutrient soil. After 1 mouth, the *Passiflora* spp. seedlings with uniform growth were selected to construct the laboratory screening model for anti-CMV activity test by mechanical friction inoculation method (Yael et al., 2006; Zhou et al., 2012). The solutions of 5% chitosan oligosaccharide AS (COS, 750-fold dilution), 30% dufulin WP (DFL, 1000-fold dilution), and 8% ningnanmycin AS (Ning, 600-fold dilution) diluted with distilled water were sprayed on the whole *Passiflora* spp. seedlings, respectively. Five *Passiflora* spp. seedlings were treated as one group, each group was repeated for three times. The distilled water was served as the blank control (CK group). After 2 days of spraying, the leaves of *Passiflora* spp. seedlings were inoculated with CMV using a brush dipped and subsequently washed with water. Then, the *Passiflora* spp. seedlings were grown in light incubators with the constant humidity of 90% at 28 °C for 14 h during the day and 26 °C for 10 h at night. After 7 days of inoculation, the leaves of *Passiflora* spp. seedlings were selected and stored at –80 °C for the subsequent laboratory anti-CMV activity experiments.

### 2.2 Laboratory anti-CMV activity test

Total RNA of the leaves of *Passiflora* spp. seedlings was extracted according to the manufacturer’s instructions of the plant RNA rapid extraction kit (ComWin Biotech Co., Ltd., Beijing, China). cDNA was reverse-transcribed according to the manufacturer’s instructions of a Goldenstar™ RT6 cDNA synthesis kit (Tsingke Biotechnology, Beijing, China). After that, the cDNAs were amplified by the polymerase chain reaction (PCR) analysis with the forward primers of 5’-ATGGACAAATCTGAATCAACCAGTGC-3’ and reverse primer of 5’-TCAGACTGGGAGCACCCCAGAC-3’. The amplification conditions were set at 94 °C for 2 min, followed by 35 cycles of 30 s at 94 °C, 30 s at 58 °C, and 70 s at 72 °C, and extended at 72°C and 12°C for 10 min, respectively. After that, the PCR products were electrophoresed on 0.75% agarose gel for 30 min to calculate the virulence rate and anti-CMV activity according to the reported method (Kang et al., 2021).

### 2.3 Field trials test

In 2021, the field trials of COS (750-fold dilution), DFL (1000-fold dilution), and Ning (600-fold dilution) against on *Passiflora* spp. CMV disease were performed in Zhenning city. Sterile distilled water was served as the blank control (CK group). On the 7th day after the first, second, and third spraying, the control efficiencies of COS, DFL, and Ning were calculated by the reported method (Montasser et al., 1998).

### 2.4 Nutritional quality test of *Passiflora* spp. fruits

To investigate the effect of COS on the nutritional quality of *Passiflora* spp. fruits, herein, the soluble solids, titratable acids, vitamin C, and soluble proteins contents of *Passiflora* spp. fruits collected on the 7th day after the third spraying were determined according to the reported methods (da Rocha et al., 2022; Xu et al., 2022; Yang et al., 2022; Zhang et al., 2022).

### 2.5 Determination of antioxidant enzyme activity

The antioxidant enzyme activities of peroxidase (POD), superoxide dismutase (SOD), and polyphenol oxidase (PPO) in the leaves of *Passiflora* spp. seedlings of COS treatment and CK groups on the 7th day after spraying were determined using the commercially available enzyme assay reagent kits (Suzhou Comin Bioengineering Institute, Suzhou, China).

### 2.6 Transcriptome sequencing

The *Passiflora* spp. seedlings with uniform growth were selected and the solution of 5% COS AS (750-fold dilution) were sprayed on the whole *Passiflora* spp. seedlings. After 7 days of spraying, the leaves of *Passiflora* spp. seedlings were collected. Transcriptome sequencing of the leaves of *Passiflora* spp. seedlings were completed by Hangzhou Lianchuan Biological Co., LTD. and sequenced using the Illumina HiSeq™ 2000 (Illumina Inc., San Diego, CA, USA). The raw data were deposit at National Center for Biotechnology Information (NCBI, https://www.ncbi.nlm.nih.gov/) database with the IDs of GSM6813002 and GSM6813001, respectively. To obtain high-quality reads, Cutadapt v1.9.3 software was used to remove the low-quality and undefined reads (Qu et al., 2021). HISAT was used to compare the reference genome of the preprocessed valid data (Kim et al., 2015). The gene expression was defined based on the FPKM value (Moon and Zhao, 2021). Salmon was used to calculate the expression level of Unigenes (Li et al., 2022a). R language package was used to identify DEGs (*p* < 0.05 and -log_2_FC > 1) (Zhang et al., 2019). Then, Gene Ontology (GO) annotation (named biological processes (BP), cellular components (CC), and molecular function (MF)) and Kyoto Encyclopedia of Genes and Genomes (KEGG) pathway enrichment for the DEGs were carried out at http://www.geneontology.org/ and http://www.genome.jp/Pathway, respectively (Yu et al., 2018).

### 2.7 Proteomics sequencing

The *Passiflora* spp. seedlings with uniform growth were selected and the solution of 5% COS AS (750-fold dilution) were sprayed on the whole *Passiflora* spp. seedlings. After 7 days of spraying, the leaves of *Passiflora* spp. seedlings were collected for proteomics analysis according to our reported method (Gao et al., 2017). The raw data were deposited to the ProteomeXchange Consortium (http://proteomecentral.proteomexchange.org) with the ID of PXD038881. The raw data were quantified by the MaxQuant software (version 1.5.8.3) (Cox et al., 2011; Yu et al., 2018). DEPs (expression level > 2.0-fold, *p* < 0.01) were identified from the Uniprot database (http://www.uniprot.org/). Then, GO annotation related at three ontologies (namely BP, CC, and MF) and KEGG pathway enrichment for the DEPs were carried out at http://www.geneontology.org/and http://www.genome.jp/Pathway, respectively (Yu et al., 2018).

### 2.8 Statistical analysis

The results of the nutritional quality in the fruits of *Passiflora* spp. and antioxidant enzyme activity in the leaves of *Passiflora* spp. seedlings were statistically analyzed using one-way ANOVA followed by LSD test using Origin 2021.

## 3 Results and discussion

### 3.1 Results of laboratory anti-CMV activity and field trials tests

Virus disease is the most common type of disease in *Passiflora* spp. and is also known as a phloem disease (Fischer and Rezende, 2008). Once infected by the virus disease, it can cause the whole plant of *Passiflora* spp. not to bloom and fruit, resulting in a continuous reduction in the yield (Carvalho et al., 2021). Unfortunately, it is well-known that the antiviral agents that are currently available on the market cannot effectively control virus disease, and the mechanism of action between antiviral agents and virus disease has not been clarified, resulting in the research on the control of *Passiflora* spp. virus disease becoming difficult. In this study, a laboratory anti-CMV activity screening model for CMV disease of *Passiflora* spp. seedlings was first established and the effects of COS, DFL, and Ning on CMV virulence rates in *Passiflora* spp. seedlings were determined and the results were listed in **Table 1**. **Table 1** showed that the virulence rate and anti-CMV activity in *Passiflora* spp. treated with COS were 50.00% and 45.48%, respectively, which were superior to those of DFL (66.67% and 27.30%, respectively) and Ning (83.30% and 9.17%, respectively), demonstrating that COS could effectively control the CMV disease in *Passiflora* spp. seedlings. Meanwhile, to study the control efficiency of COS, DFL, and Ning on the control of CMV disease of *Passiflora* spp., a preliminary study on the control of *Passiflora* spp. CMV disease was conducted through field efficacy trials. As shown in **Table 2**, on the 7th day after the first, second and third spraying, COS was effective in reducing *Passiflora* spp. CMV disease in the field (the average control efficiency is 47.35%) relative to those of DFL (40.93%) and Ning (33.82%). In the past few years, many studies had demonstrated that COS revealed good antiviral activity. Yu et al. (2002) and Li et al. (2019) reported that COS had obvious control efficiency on CMV disease in cucumber and tobacco. There are fewer studies related to the control of *Passiflora* spp. virus disease with antiviral agents. Kitajima et al. (2003) and Crestani et al. (1986) reported the first outbreak of *Passiflora* spp. virus disease in Brazil, and it was demonstrated experimentally that the incidence of the disease could be significantly reduced by the following antiviral agents hexythiazox, quinomethionate, dicofol, propargite, or fenbutatin-oxide.

**Table 1:** Results of Laboratory Anti-CMV Activity of COS, DFL, and Ning.

Treatments	Virulence rate (%)*	Anti-CMV activity (%)*
CK	91.70 ± 2.16a	–
COS	50.00 ± 1.25d	45.48 ± 2.24a
DFL	66.67 ± 2.13c	27.30 ± 1.26b
Ning	83.30 ± 3.11b	9.17 ± 1.56c

*Different lowercase letters indicated the disease index and control efficiency of COS treatment group with significant difference compared with CK group (p < 0.05).

**Table 2:** The control efficiencies of COS, DFL, and Ning against *Passiflora* spp. CMV disease.

Treatments	7 Days after the first spraying		7 Days after the second spraying		7 Days after the third spraying		Average control efficiency (%)*
	Disease index (%)*	Control efficiency (%)*	Disease index (%)*	Control efficiency (%)*	Disease index (%)*	Control efficiency (%)*	
CK	12.50 ± 0.34a	–	27.78 ± 0.34a	–	46.57 ± 0.45a	–	–
COS	8.23 ± 0.20cd	34.16 ± 0.12a	13.60 ± 0.32d	51.04 ± 1.33a	20.09 ± 0.23d	56.86 ± 0.43a	47.35 ± 0.55a
DFL	8.60 ± 0.02c	31.20 ± 0.30b	16.39 ± 0.66c	41.00 ± 0.93b	23.01 ± 0.90c	50.59 ± 0.23b	40.93 ± 1.23b
Ning	9.72 ± 0.09b	22.24 ± 0.19c	18.70 ± 0.50b	32.69 ± 0.99c	24.89 ± 0.45b	46.55 ± 0.74c	33.82 ± 0.97c

*Different lowercase letters indicated the disease index and control efficiency of different treatment group with significant difference compared with CK group (p < 0.05).

### 3.2 Results of nutritional quality of *Passiflora* spp. fruits

**Figure 1:**
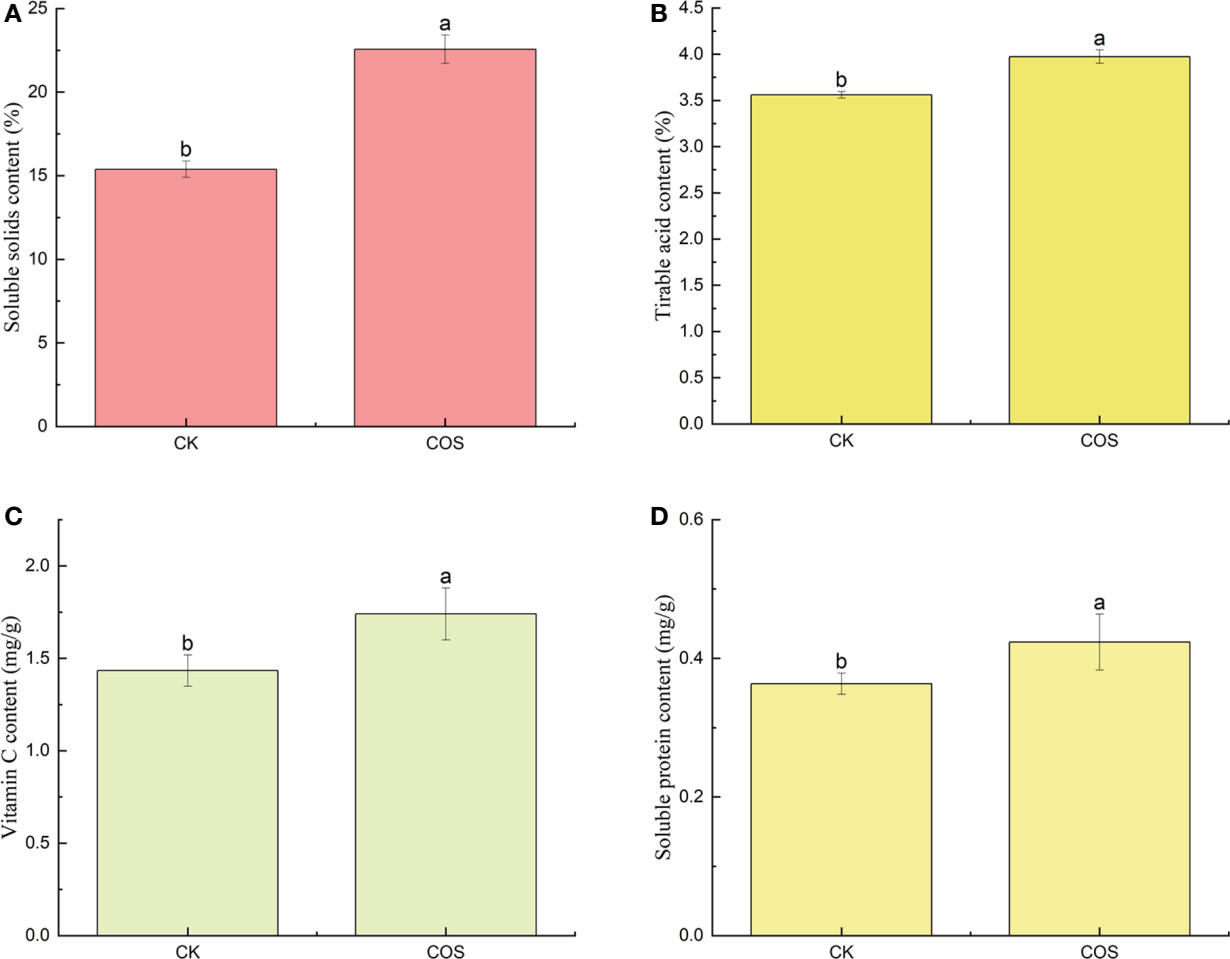
Effect of COS on the soluble solids **(A)**, titratable acids **(B)**, vitamin C **(C)**, and soluble proteins **(D)** contents of *Passiflora* spp. fruits. Different lowercase letters indicated the effect of COS on the soluble solids, titratable acids, vitamin C, and soluble proteins contents of Passiflora spp. fruits with significant difference compared with CK group (p < 0.05).

In recent years, as the people attach increasing importance to their health, the nutritional quality of fruits has been widely requested by consumers (Wang et al., 2020). As known to all, nutritional quality indexes, such as soluble solids, titratable acids, vitamin C, and soluble proteins contents, are the most important factors on fruit quality, the taste and flavor of fruits, and the consumer purchasing decision (Huang et al., 2021). Soluble solids content, an important index of fruit ripening process and economic benefits, is associated with fruit taste and harvest time (Li et al., 2016; Han et al., 2017). As shown in **Figure 1A**, COS could increase the content of soluble solids in *Passiflora* spp. fruits. Titratable acids are the main index affecting fruit taste and the substrate of respiration metabolism (Guo et al., 2022). After COS treatment, as shown in **Figure 1B**, the increase of titratable acids content could a certain extent improve the *Passiflora* spp. fruits respiration. Vitamin C, one of the nutritional components, is an important antioxidant in fruits to reduce the damage to cell membranes (Nair et al., 2018). **Figure 1C** showed that COS treatment could significantly increase the vitamin C content in *Passiflora* spp. fruits which not only improved the nutritional quality of fruit, but also increased the disease resistance. Soluble proteins might be attributed to hydrolysis of cell membranes induced by ripening. Reactive oxygen species (ROS) damage and fungal infection could cause the decrease trend of soluble protein in the fruit (Scandalios, 1993; Alia-Tejacal et al., 2007). **Figure 1D** showed that COS could increase the content of soluble proteins. To sum up, COS could improve the taste and flavor of *Passiflora* spp. fruits. Similar results were also reported by He et al. (2018), they found that COS could significantly increase the contents of soluble solids, titratable acids, vitamin C, and soluble proteins in strawberry.

### 3.3 Effect on the antioxidant enzyme activity

**Figure 2:**
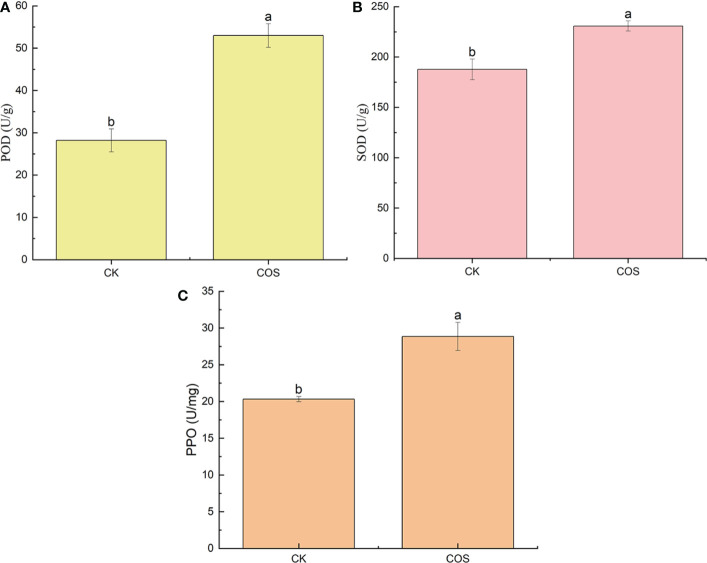
Effect of COS on the POD **(A)**, SOD **(B)**, and PPO **(C)** activity in the leaves of *Passiflora* spp. seedlings. Different lowercase letters indicated the effect of COS on the POD, SOD, and PPO activity in the leaves of Passiflora spp. seedlings with significant difference compared with CK group (p < 0.05).

Plant disease resistance is associated with the activation of a series of defense responses, such as induce defense responses in the form of enzymes, that slow down or stop infection during certain stages of host-pathogen interaction. POD can induce the biosynthetic pathway of salicylic acid (SA) to activate the systemic acquired resistance (SAR), thus enhancing the tolerance to pathogenic microorganism infection (Wu et al., 2013; Yang et al., 2019). SOD is a key enzyme of oxygen metabolism in plants, which plays a critical role in eliminating superoxide free radicals, alleviating lipid peroxidation and membrane damage under stress (Díaz et al., 2001). PPO activity plays an important role in plant defense mechanisms which can help the plant resist adversity and develop adaptive strategies to enhance plant resilience (Taranto et al., 2021). In this study, to clarify the effect of COS on the antioxidant enzyme activity in the leaves of *Passiflora* spp. seedlings, the POD, SOD, and PPO activity were studied. As shown in **Figure 2**, after 7 days of spraying, COS could increase the POD, SOD, and PPO activity in the leaves of *Passiflora* spp. seedlings to activate the antioxidant defense system in *Passiflora* spp. seedlings against CMV disease. Liu et al. (2021) reported that COS could significantly increase the antioxidant enzyme (POD, SOD, and PPO) activity to promote the growth of wheat. Li et al. (2022b) found that the application of physcion and COS combination is more effective in facilitating the SOD, POD activities of the maize plants, resulting in plants with high stress resistance. Ou et al. (2022) reported that COS could enhance the antioxidant activity of SOD and POD in tea plants.

### 3.4 Quality check of transcriptome sequencing data

The cDNA libraries of the leaves of *Passiflora* spp. seedlings of COS treatment and CK groups were characterized using Illumina HiSeq platform to detect the transcriptome level of gene expression information according to the reported methods (Chen et al., 2014; Wang et al., 2022). The data obtained by up-sequencing were removed from sequencing junctions and low-quality, and the reads with unidentifiable base information and the proportion of more than 5% were filtered out. The original sequencing volume (raw data), effective sequencing volume (valid data), as well as the contents of Q20, Q30 and GC were counted and the summary results were displayed in **Table 3**. **Table 3** showed that the transcriptome sequencing data were of high quality for the subsequent bioinformatics analysis with the valid ratios for both samples > 92%, the proportions of Q20 and Q30 bases for both samples > 97%, and the GC contents for both samples > 44%.

**Table 3:** The summary quality statistics results of the transcriptome sequencing data.

Samples	Raw data	Valid data	Valid ratio (%)	Q20 (%)	Q30 (%)	GC (%)
CK	51942238	47952536	92.32	99.99	97.92	44.50
COS	51529044	49747664	96.54	99.99	97.80	44.00

### 3.5 Results of DEGs and bioinformatic analysis

**Figure 3:**
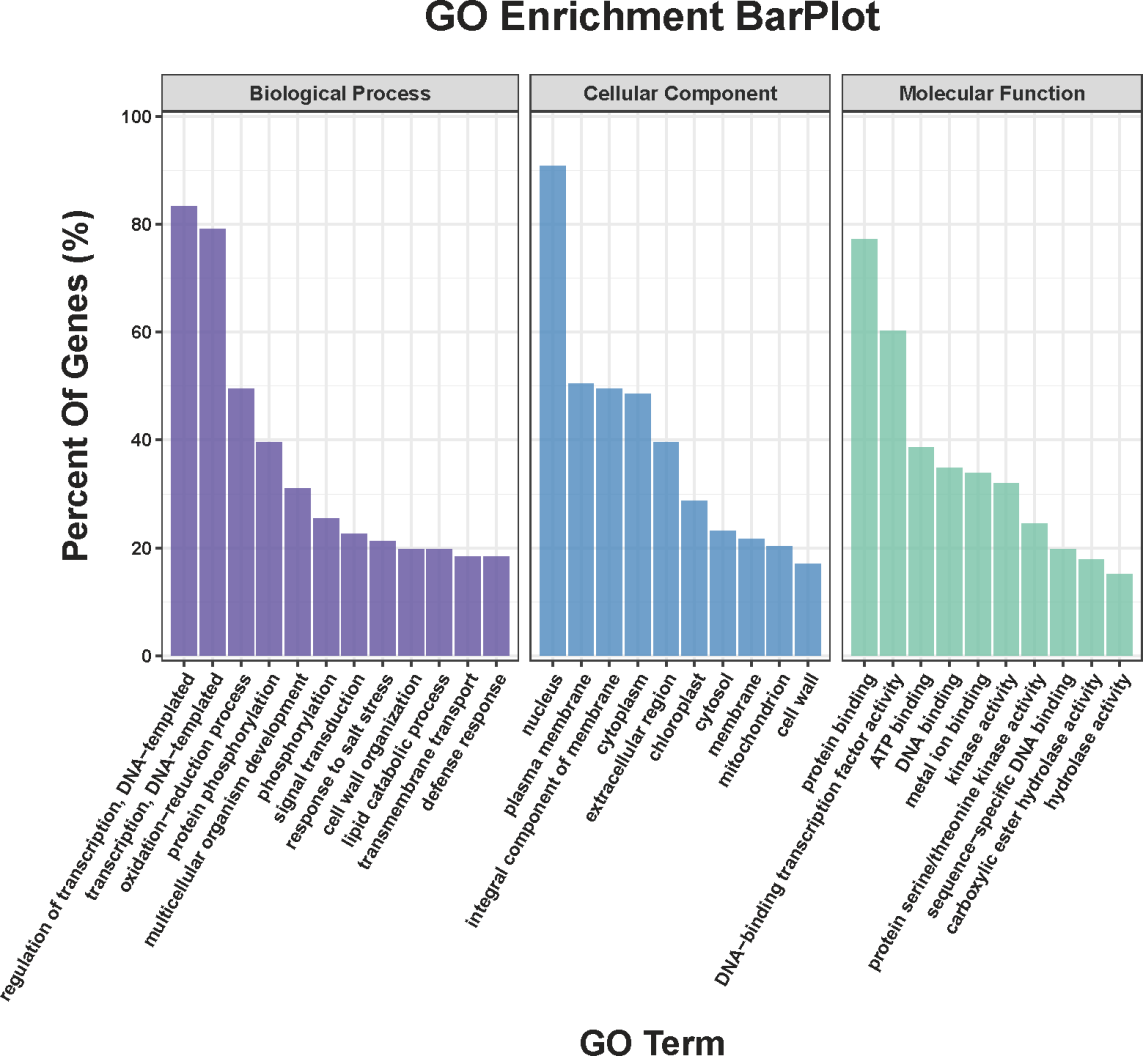
GO classification of DEGs.

Compared COS treatment group with CK group, a total of 911 DEGs were obtained in the transcriptome sequencing (**Table S1**), of which, the up- and down-regulated genes were 557 and 354, respectively. Meanwhile, **Figure 3** showed that DEGs between the COS treatment and CK groups were analyzed for GO categories in BP (regulation of transcription, DNA-templated (GO:0006355), transcription, DNA-templated (GO:0006351), oxidation-reduction process (GO:0055114), protein phosphorylation (GO:0006468), multicellular organism development (GO:0007275), phosphorylation (GO:0016310), signal transduction (GO:0007165), response to salt stress (GO:0009651), cell wall organization (GO:0071555), and lipid catabolic process (GO:0016042)), CC (nucleus (GO:0005634), plasma membrane (GO:0005886), integral component of membrane (GO:0016021), cytoplasm (GO:0005737), extracellular region (GO:0005576), chloroplast (GO:0009507), cytosol (GO:0005829), membrane (GO:0016020), mitochondrion (GO:0005739), and cell wall (GO:0005618)), and MF (protein binding (GO:0005515), DNA-binding transcription factor activity (GO:0003700), ATP binding (GO:0005524), DNA binding (GO:0003677), metal ion binding (GO:0046872), kinase activity (GO:0016301), protein serine/threonine kinase activity (GO:0004674), sequence-specific DNA binding (GO:0043565), carboxylic ester hydrolase activity (GO:0052689), and hydrolase activity (GO:0016787)).

**Figure 4:**
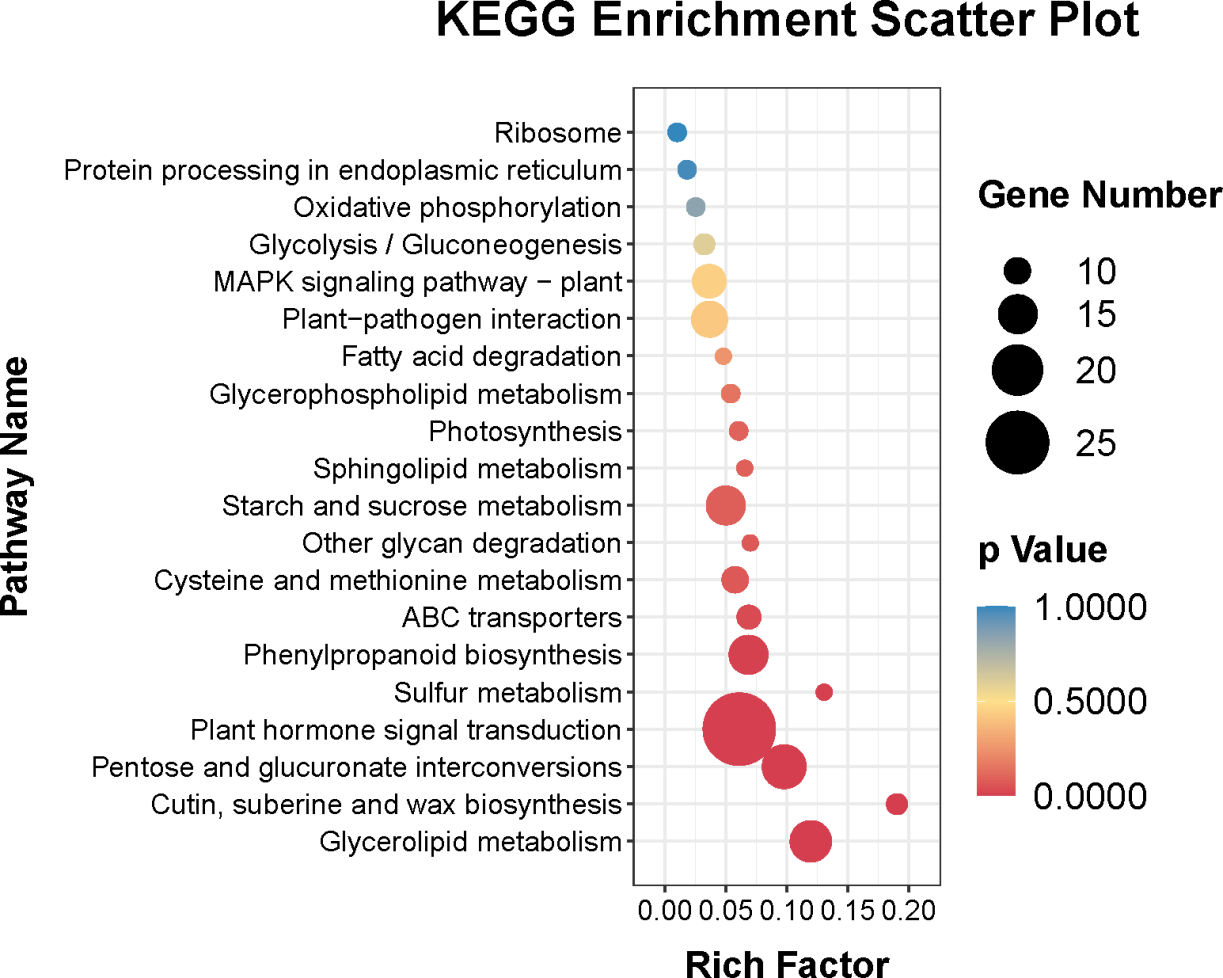
Top twenty KEGG pathways enrichment of DEGs.

In addition, **Figure 4** showed that the top 20 enriched KEGG pathways of the DEGs were plant hormone signal transduction (ko04075), pentose and glucuronate interconversions (ko 00040), glycerolipid metabolism (ko00561), phenylpropanoid biosynthesis (ko00940), starch and sucrose metabolism (ko00500), plant-pathogen interaction (ko04626), MAPK signaling pathway–plant (ko04016), cysteine and methionine metabolism (ko00270), ABC transporters (ko02010), cutin, suberine and wax biosynthesis (ko00073), glycolysis/gluconeogenesis (ko00010), photosynthesis (ko00195), glycerophospholipid metabolism (ko00564), oxidative phosphorylation (ko00190), protein processing in endoplasmic reticulum (ko04141), ribosome (ko03010), sulfur metabolism (ko00920), other glycan degradation (ko00511), sphingolipid metabolism (ko00600), and fatty acid degradation (ko00071).

### 3.6 Results of DEPs and bioinformatic analysis

**Figure 5:**
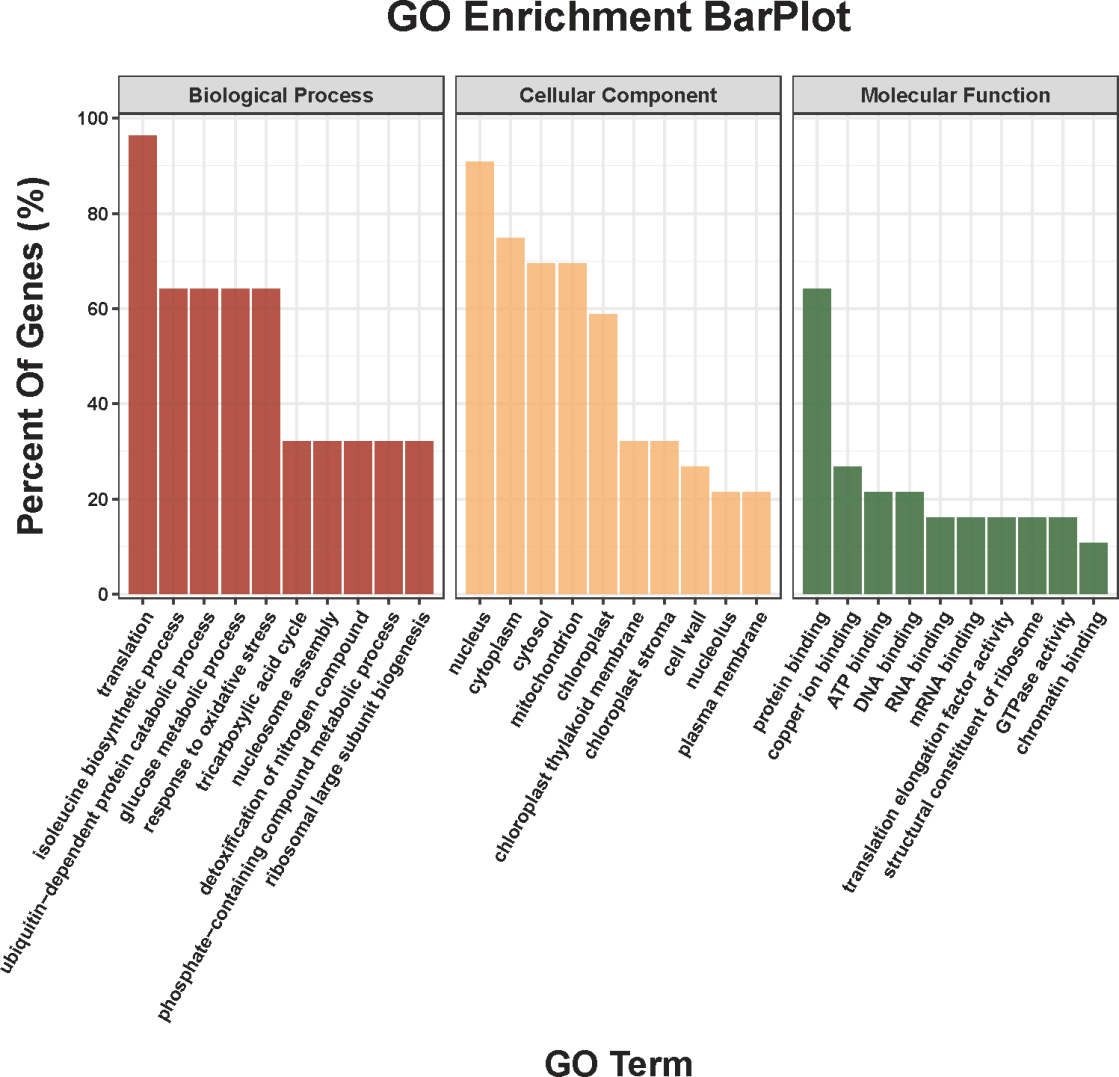
GO classification of DEPs.

A total of 667 proteins were identified, of which, the up- and down-regulated proteins were 42 and 30, respectively, in COS treatment group compared to the CK group (**Table S2**). Meanwhile, as shown in **Figure 5**, the GO term enrichment analysis revealed that the DEPs were mainly involved in the following enrichment categories: translation (GO:0006412), isoleucine biosynthetic process (GO:0009097), ubiquitin-dependent protein catabolic process (GO:0006511), glucose metabolic process (GO:0006006), and response to oxidative stress (GO:0006979) in terms of BP category; ten categories, namely, nucleus (GO:0005634), cytoplasm (GO:0005737), cytosol (GO:0005829), mitochondrion (GO:0005739), chloroplast (GO:0009507), chloroplast thylakoid membrane (GO:0009535), chloroplast stroma (GO:0009570), and cell wall (GO:0005618) in terms of CC category; ten categories, namely, protein binding (GO:0005515), copper ion binding (GO:0005507), ATP binding (GO:0005524), DNA binding (GO:0003677), RNA binding (GO:0003723), mRNA binding (GO:0003729), translation elongation factor activity (GO:0003746), structural constituent of ribosome (GO:0003735), and GTPase activity (GO:0003924) in terms of MF category.

**Figure 6:**
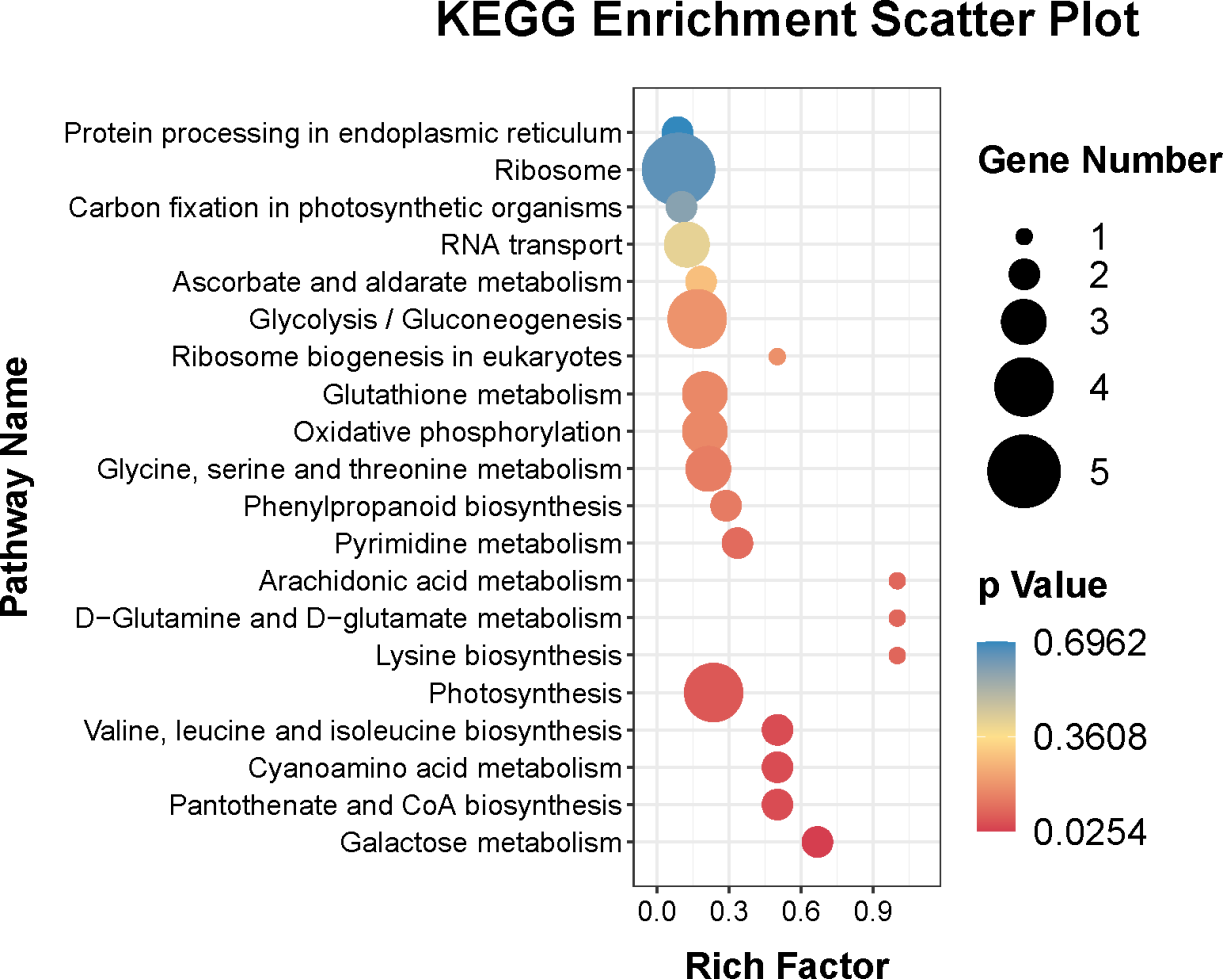
Top twenty KEGG pathways enrichment of DEPs.

In addition, the KEGG pathways of the DEPs were also identified. **Figure 6** showed that the top twenty KEGG pathways were ribosome (ko03010), photosynthesis (ko00195), glycolysis/gluconeogenesis (ko00010), glycine, serine and threonine metabolism (ko00260), glutathione metabolism (ko00480), oxidative phosphorylation (ko00190), RNA transport (ko03013), galactose metabolism (ko00052), valine, leucine and isoleucine biosynthesis (ko00290), cyanoamino acid metabolism (ko00460), pantothenate and CoA biosynthesis (ko00770), pyrimidine metabolism (ko00240), phenylpropanoid biosynthesis (ko00940), ascorbate and aldarate metabolism (ko00053), carbon fixation in photosynthetic organisms (ko00710), and protein processing in endoplasmic reticulum (ko04141).

### 3.7 Combined analysis of transcriptomic and proteomic technology

**Figure 7:**
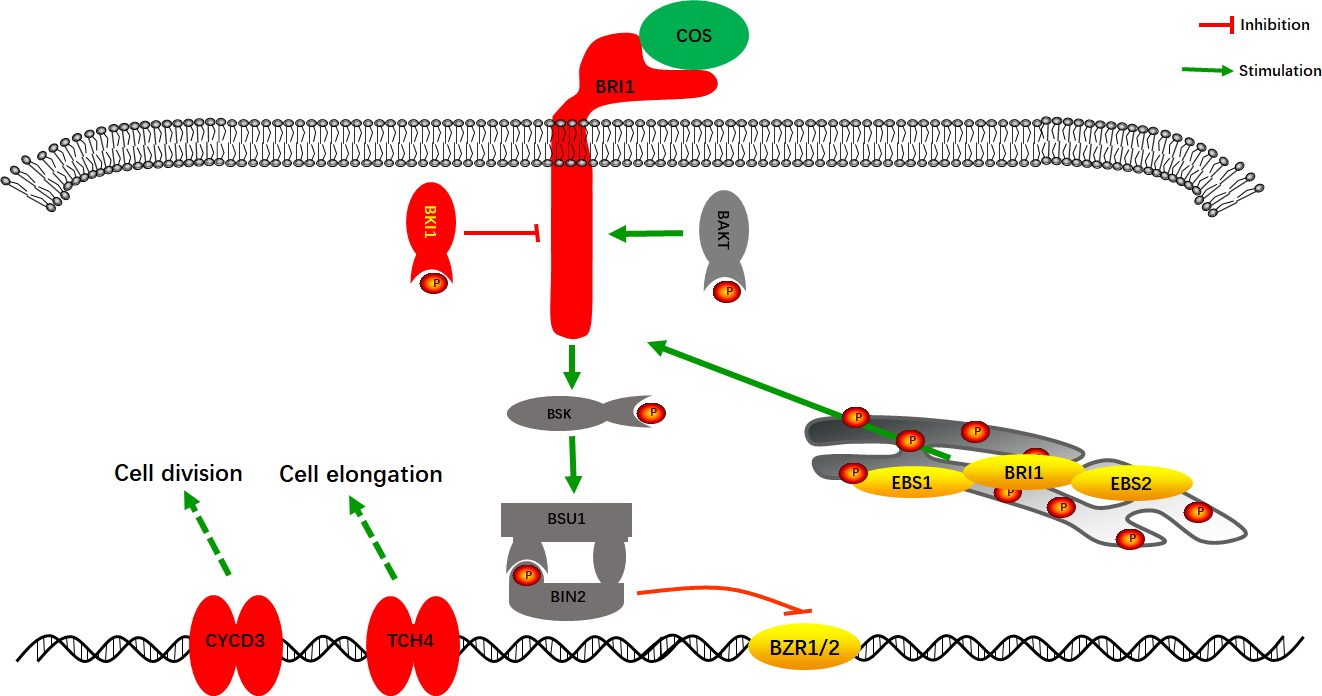
A model of the BRs cell signaling pathway in *Passiflora* spp. response to COS. Red, up-regulated expression; Yellow, down-regulated expression; P, phosphorylation.

The combined transcriptome and proteome analysis results showed that COS mainly affected the Brassinosteroids (BRs) cell signaling pathway in *Passiflora* spp. to improve the resistance to CMV disease. BRs cell signaling pathway is a class of plant hormone signal transduction, which plays diverse roles in plant growth and development (Hecht et al., 2001; Li et al., 2002; Zipfel, 2008; Yang et al., 2011). As shown in **Figure 7**, after COS treatment, the gene expression of cell membrane surface receptor kinase *Brassinosteroid insensitive 1* (*BRI1*) was stimulated, and the gene and protein expression of its negative regulatory protein BRI1 kinase inhibitor 1 (BKI1) were up-regulated, thus inhibiting the BRs cell signaling pathway and then making it unable to complete phosphorylation signal transduction to form *Brassinosteroid insensitive 2* (*BIN2*) to inhibit the transcription factor *BZR1* and *BZR2* (*BZR1/2*). After that, *BZR1/2* could bind to specific regions on promotors of the downstream target genes to activate the up-regulated expression of *TCH4* and *CYCD3* genes to improve the disease resistance. *TCH4* gene plays an important role in the biological processes such as cell elongation, resistance to disease, and adaptation to environmental stress in plants (Zhang et al., 2008; Friedrichsen and Chory, 2001; Xu et al., 2001). Studies have demonstrated that *CYCD3* gene, involved in the BRs cell signaling pathway, is involved in cell division and may play an important role in blocking pathogen infection by regulating cell wall remodeling in the early stage of resistant varieties (Hu et al., 2000; Yan et al., 2021).

## 4 Conclusions

In conclusion, our study results demonstrated that COS revealed an effective control efficiency against CMV disease of *Passiflora* spp. and could be used as a potential plant immune inducer by mainly acting on the BRs cell signaling pathway in *Passiflora* spp. to improve the resistance to CMV disease. Therefore, this study lays the foundation for a more in-depth study of the molecular mechanism of disease resistance in *Passiflora* spp. and provides a theoretical basis for application effect of COS as a plant immune inducer on the *Passiflora* spp. CMV disease.

## Data availability statement

The datasets presented in this study can be found in online repositories. The names of the repository/repositories and accession number(s) can be found in the article.

## Author contributions

Methodology, LZ and ZZ. Data analysis, LZ and ZZ. Writing—review and editing, LY, PL, and ST. Funding acquisition, LY and PL. All authors contributed to the article and approved the submitted version.
